# End-Tidal Carbon Dioxide Pressure Measurement after Prolonged Inspiratory Time Gives a Good Estimation of the Arterial Carbon Dioxide Pressure in Mechanically Ventilated Patients

**DOI:** 10.3390/diagnostics11122219

**Published:** 2021-11-27

**Authors:** Arthur Salomé, Annabelle Stoclin, Cyrus Motamed, Philippe Sitbon, Jean-Louis Bourgain

**Affiliations:** 1 Assistance Publique des Hôpitaux de Paris, 94270 Le Kremlin Bicêtre, France; arthur.salome1995@gmail.com; 2 Faculty of Medicine, University of Paris-Saclay, 94270 Le Kremlin Bicêtre, France; 3 Intensive Care Unit, Gustave Roussy Institute, 94800 Villejuif, France; annabelle.stoclin@gustaveroussy.fr; 4 Anesthesia Department, Gustave Roussy Institute, 94800 Villejuif, France; cyrus.MOTAMED@gustaveroussy.fr (C.M.); philippe.sitbon@gustaveroussy.fr (P.S.)

**Keywords:** carbon dioxide monitoring, hypercapnia detection, COVID-19, ARDS, mechanical ventilation, CO_2_ gradient

## Abstract

Background: End-tidal carbon dioxide pressure (PetCO_2_) is unreliable for monitoring PaCO_2_ in several conditions because of the unpredictable value of the PaCO_2_–PetCO_2_ gradient. We hypothesised that increasing both the end-inspiratory pause and the expiratory time would reduce this gradient in patients ventilated for COVID-19 with Acute Respiratory Distress Syndrome and in patients anaesthetised for surgery. Methods: On the occasion of an arterial blood gas sample, an extension in inspiratory pause was carried out either by recruitment manoeuvre or by extending the end-inspiratory pause to 10 s. The end-expired PCO_2_ was measured (expiratory time: 4 s) after this manoeuvre (PACO_2_) in comparison with the PetCO_2_ measured by the monitor. We analysed 67 Δ(a-et)CO_2_, Δ(a-A)CO_2_ pairs for 7 patients in the COVID group and for 27 patients in the anaesthesia group. Results are expressed as mean ± standard deviation. Results: Prolongation of the inspiratory pause significantly reduced PaCO_2_–PetCO_2_ gradients from 11 ± 5.7 and 5.7 ± 3.4 mm Hg (*p* < 0.001) to PaCO_2_–PACO_2_ gradients of −1.2 ± 3.3 (*p* = 0.043) and −1.9 ± 3.3 mm Hg (*p* < 0.003) in the COVID and anaesthesia groups, respectively. In the COVID group, PACO_2_ showed the lowest dispersion (−7 to +6 mm Hg) and better correlation with PaCO_2_ (R^2^ = 0.92). The PACO_2_ had a sensitivity of 0.81 and a specificity of 0.93 for identifying hypercapnic patients (PaCO_2_ > 50 mm Hg). Conclusions: Measuring end-tidal PCO_2_ after prolonged inspiratory time reduced the PaCO_2_–PetCO_2_ gradient to the point of obtaining values close to PaCO_2_. This measure identified hypercapnic patients in both intensive care and during anaesthesia.

## 1. Introduction

Lung involvement during SARS-CoV-2 infection frequently dominates the clinical picture of severe forms with both interstitial pneumonia related to the release of pro-inflammatory cytokines and vascular obstruction of thromboembolic origin [1]. According to international recommendations for Acute Respiratory Distress Syndrome (ARDS) management, protective ventilation is the rule. This practice often induces hypercapnia and requires regular checks of arterial CO_2_ pressure (PaCO_2_) to adjust the level of ventilation.

In healthy anaesthetised patients, the monitoring of end-tidal CO_2_ pressure (PetCO_2_) is mandatory even if it underestimates the PaCO_2_, with a gradient around 4.5–13 mm Hg [2]. Lung diseases are associated with pulmonary heterogeneity, and the PaCO_2_–PetCO_2_ gradient rises in unpredictable proportions [3,4,5,6,7,8]. Therefore, the PetCO_2_ value cannot be taken as a good estimate of alveolar PCO_2_ and hence cannot be used as an estimate of PaCO_2_. In previous studies in patients with acute lung injury, longer inspiratory time without a change in respiratory rate enhanced CO_2_ exchange [8]. During controlled ventilation in adults, the “expiratory plateau” is usually flat, but several factors, such as age and pulmonary disease, are associated with an increase in the slope of the “expiratory plateau”. A prolonged expiration manoeuvre improves the prediction of PaCO_2_ from end-tidal PCO_2_ [9].

Our hypotheses were that end-tidal PCO_2_ measured immediately after an inspiratory time (PACO_2_) prolonged by an inspiratory pause or recruitment manoeuvre could improve the estimation of PaCO_2_ and identify severe hypercapnia during mechanical ventilation in COVID ARDS patients or in patients anaesthetised for major surgery.

## 2. Methods

This study complied with the National Medical Ethics Regulation and was approved by the institution’s Clinical Research Commission on May 2020. Patients or relatives received information that their anonymous data would be used for research purposes and did not object.

### 2.1. Study Design

This study was prospective and observational, involving routine care of a unicentric cohort, and conducted at Gustave Roussy, Villejuif, France, in both the ICU and operating theatre. Included were patients with a COVID-19 diagnosis hospitalised for ARDS (COVID group), according to the Berlin definition [10], and patients anaesthetised for major cancer surgery requiring invasive arterial monitoring (anaesthesia group). Patients under protective mechanical ventilation were deeply anaesthetised, with or without muscle relaxation. Increased inspiratory time was used in routine care in two situations: recruitment manoeuvre or prolongation of end-inspiratory pause to measure static compliance. In both groups, measurements were performed only if a bad tolerance of apnoea or recruitment was not predicted because of haemodynamic conditions, and if it would not hinder the surgeon.

### 2.2. Protocol

In both groups, a 10 s (arbitrarily fixed) period of apnoea was obtained using either the end-inspiratory pause function for static compliance evaluation or an automatic recruitment manoeuvre on the intensive care respirator (Evita) or Perseus anaesthesia respirator. With the Zeus anaesthesia respirator, the manoeuvre consisted of sustained manual inflation of the anaesthesia reservoir bag to a peak inspiratory pressure of 30 cm H_2_O for 10 s. The fraction of inspired oxygen concentration (FiO_2_) was not changed during the procedures. Expiration time was set to 4 s by adjusting the respiratory rate to 10 cycles per minute, and an I:E ratio around 1:2, to obtain an expiratory plateau on CO_2_ recording. PCO_2_ was measured at the end of the 4 s expiration following the inspiratory pause or recruitment manoeuvre (PACO_2_).

The same investigator carried out all measurements, using a side-stream CO_2_ sensor, to ensure their comparability.

An arterial blood gas measurement was collected prior to each measurement of PACO_2_, to calculate the PaCO_2_–PetCO_2_ and the PaCO_2_–PACO_2_ gradients and the PaO_2_/FiO_2_ ratio. All patients already needed arterial catheters: none were placed expressly for the study.

As the patients’ respiratory and haemodynamic status varied over time, several measurements could be conducted for the same patient.

### 2.3. Other Collected Data

FiO_2_, basal EtCO_2_, tidal volume (Vt), respiratory rate (RR), inspiratory plateau pressure (Pplate), settled positive end expiratory pressure (PEEP), central temperature, and when available, level of muscle relaxation (T4/T1), haemodynamic profile including the heart rate (bpm), mean arterial pressure (mm Hg), and norepinephrine dose (milligrams per hour), were recorded for each measurement.

### 2.4. Statistical Analysis

Data are presented as mean values ± standard deviations. PaCO_2_ values were temperature-corrected, according to the Siggaard-Andersen equation [11]. The normal distributions of PetCO_2_, PACO_2_, and PaCO_2_ data were assessed using the Q-Q plot method.

When PACO_2_ was available from both the inspiratory pause and recruitment, the first measurement was considered for gradient analyses. Indeed, a residual effect of the first recruitment may bias the second measurement.

A paired, two-tailed Student’s *t*-test was used to compare the different values of PCO_2_. Correlation coefficients were obtained using the Pearson method to assess the correlation between pairs of PaCO_2_, PetCO_2_, and PACO_2_. PaCO_2_–PetCO_2_, PaCO_2_–PACO_2_, and PACO_2_–PetCO_2_ gradient data were compared using a two-tailed unpaired *t*-test.

Bland–Altman plots were used to test for paired PetCO_2_, PACO_2_, and PaCO_2_ agreement and reported with the 95% and 99% confidence intervals, for the separate and combined groups.

The statistical performance of PACO_2_ to detect normocapnia and hypercapnia was calculated for different thresholds of PACO_2_. It was calculated for the separate and combined groups.

All statistical results were considered significant if the *p*-value was less than 0.05. All calculations were performed using Microsoft Excel data processing software (2019 version).

## 3. Results

### 3.1. Population

From 8 April to 28 May 2020, 67 PACO_2_ were obtained for 34 patients (35 measures in 7 patients in the COVID group; 32 measures in 27 patients in the anaesthesia group).

Table 1 shows demographic data and baseline characteristics of the study day. Data were similar, except for PaCO_2_, with more hypercapnia in the COVID group (21 vs. 10), as expected. The COVID group presented worse pulmonary characteristics, with a lower compliance (24 vs. 71 mL cm H_2_O^–1^ in the anaesthesia group), and a lower PaO_2_/FiO_2_ ratio (264 vs. 337 mm Hg).

In the COVID group, only 29 basal PetCO_2_ values were available, due to an initial protocol breach (6 missing data for 2 patients). No PaCO_2_ and no PACO_2_ values were missing. Five patients were ventilated using an Evita intensive care respirator (corresponding to twelve measurements), and two patients by use of a Perseus anaesthesia respirator, due to a shortage of Evita respirators in the COVID context. In the anaesthesia group, no PetCO_2_, PACO_2_, or PaCO_2_ values were missing.

### 3.2. Student’s t-Tests

PetCO_2_ underestimated PaCO_2_ in both groups, with a mean level of Δ(a-et)CO_2_ of +11 ± 5.7 mm Hg (95% CI 8.8–13) and +5.7 ± 3.4 mm Hg (4.4–6.9) in the COVID and anaesthesia group, respectively (*p* < 0.001 for both). Increased inspiratory pause or recruitment manoeuvres significantly reduced the gradient to −1.2 ± 3.3 mm Hg (−2.3 to −0.05) (*p* = 0.043) in the COVID group, and −1.9 ± 3.3 mm Hg (−3.1 to −0.07) (*p* < 0.003) in the anaesthetised patients, with no significant difference between the two groups. Table 2.

Both PetCO_2_ and PACO_2_ showed statistically significant linear correlation with PaCO_2_ for the two groups. In both the separate groups and combined groups, increased inspiratory pause or recruitment manoeuvres improved this correlation. COVID–PACO_2_ showed the best correlation (R^2^ = 0.92).

### 3.3. Bland–Altman Dispersion

**Figure 1:**
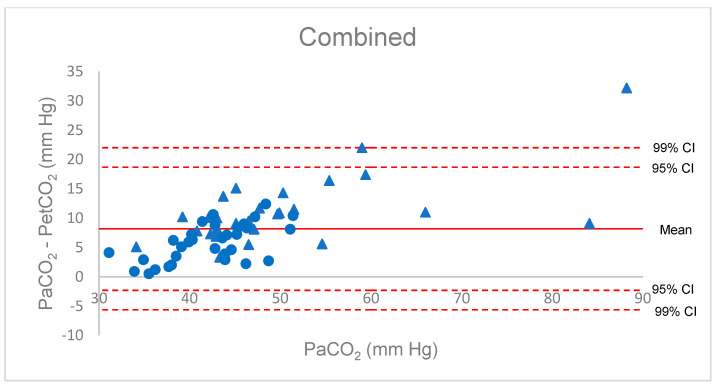
Bland–Altman diagram for the P(a-et)CO_2_ gradient, combined group. Red triangles: COVID patients; blue circles: anaesthesia patients.

**Figure 2:**
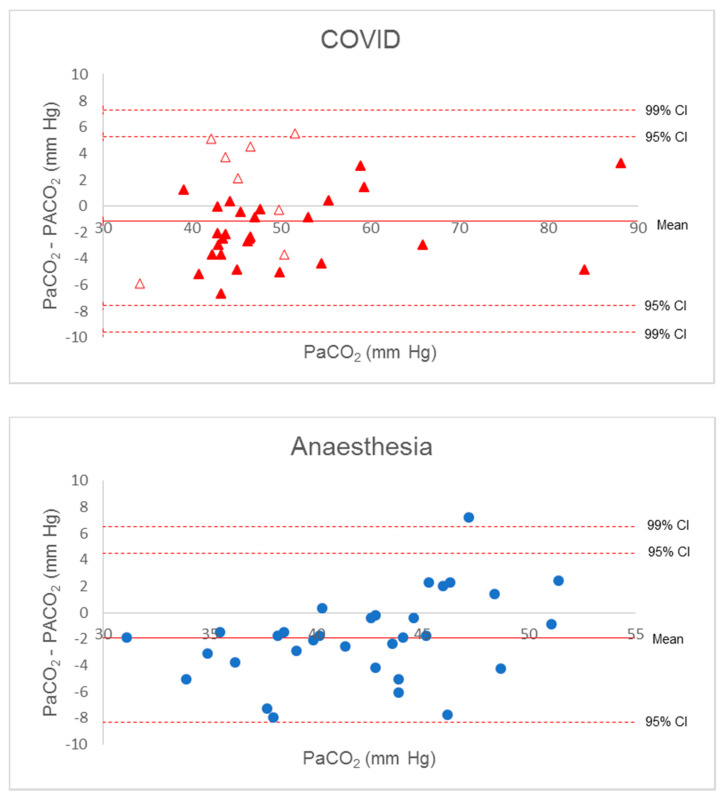

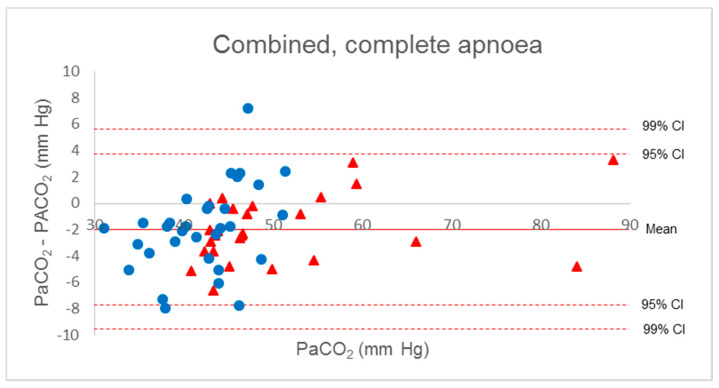
Bland–Altman diagram for P(a-A)CO_2_ gradient. Solid red triangles, complete apnoea COVID patients; empty red triangles, incomplete apnoea COVID patient; blue circles, anaesthesia patients.

The Bland–Altman diagram for Δ(a-et)CO_2_ confirmed the poor estimation of PaCO_2_ by PetCO_2_. Considering all patient data, all but two values were within a large 95% confidence interval (−2 to +19), and the gradient increased with PaCO_2_, with great imprecision beyond 50 mm Hg (Figure 1). Increased inspiratory pause or recruitment manoeuvre induced a decrease in Δ(a-A)CO_2_, with a lower dispersion from −8 to +8 mm Hg. All but one value (from the anaesthesia group) were in the 99% confidence interval around the mean of the differences. These findings were verified when considering the separate groups (Figure 2).

Five measurements in the COVID group had a gradient higher than +2 mm Hg associated with suspected respiratory movements identified on the respiratory flow curves. Keeping only the measurements on apnoeic patients, the average and 99% confidence interval of Δ(a-A)CO_2_ decreased slightly (mean −2.1 ± 2.5 mm Hg (−3.1 to −1.0)).

All patients with a PACO_2_ greater than 50 mm Hg were hypercapnic, with a PaCO_2_ higher than 45 mm Hg. For both COVID and anaesthetised patients, the PACO_2_ threshold of 48 mm Hg yielded the best performance in identifying hypercapnic patients, with Sensitivity (Se) = 0.68, Specificity (Sp) = 0.92, Positive Predictive Value (PPV) = 0.88, Negative Predictive Value (NPV) = 0.77, positive Likelihood Ratio (LR+) = 8.1, and negative Likelihood Ratio (LR−) = 0.35. The global performance improved in the COVID group, with Se = 0.81, Sp = 0.93, PPV = 0.94, NPV = 0.77, LR+ = 11, and LR− = 0.21.

## 4. Discussion

Measuring PCO_2_ at the end of expiration following a recruitment manoeuvre or an inspiratory pause significantly decreased the gradient Δ(a-et)CO_2_ in both COVID and anaesthetised patients. The correlation between PACO_2_ and PaCO_2_ was better than the correlation between PetCO_2_ and PaCO_2_, especially in the COVID group. The measurement of PACO_2_ after inspiratory pause is completely non-invasive and does not require the patient to disconnect. It nevertheless justifies hygiene precautions such as those commonly observed during the management of suspected, probable, and confirmed cases of COVID-19 [12].

In the presence of correct measurement conditions, the absence of leakage and a respiratory rate < 30 per minute, PetCO_2_ allows the estimation of PaCO_2_ in healthy subjects in spontaneous ventilation [13]. Under anaesthesia, especially in mechanical ventilation, the Δ(a-et)CO_2_ cannot be overlooked. This gradient varies from patient to patient and increases with age, smoking, ASA class, lung disease (especially in cases of pulmonary embolism), and bradycardia. Δ(a-et)CO_2_ is not stable during anaesthesia, ranging from 4.5 to 13 mm Hg [2,14].

In the ARDS, the Δ(a-et)CO_2_ tends to increase with lung heterogeneity, as shown by Yousuf and colleagues in 2017: a greater gradient in moderate vs. mild ARDS was reported but no significant difference was found in severe vs. moderate ARDS [15]. A possible explanation could be that severe ARDS was associated with pulmonary hypertension and lower cardiac output, increasing dead-space areas that attenuate the gradient.

Many different methods have been used to estimate the PaCO_2_. PetCO_2_ presents a weak correlation in healthy lungs [14,16,17,18,19,20], worsening in sick lungs, with no difference in the accuracy using a main- or side-stream sensor [21,22]. In diseased lungs, results vary greatly between studies, with moderate correlations and large dispersion around the reference PaCO_2_ value [8,15]. While the measurement of transcutaneous PCO_2_ yields better results, its use is essentially limited to the paediatric intensive care unit [23].

Inspiratory time influences the PetCO_2_ value [24]. Some manoeuvres, such as a simple prolonged exhalation, have been proposed to reduce P(a-et)CO_2_ [9].

Out of a series of 16 patients undergoing thoraco-abdominal oesophagectomy, Tavernier and colleagues [9] showed a decrease in the P(a-et)CO_2_ gradient after prolonged expiration or prolonged expiration preceded by lung hyperinflation, from 9.8 ± 3.0 to 6.0 ± 3.8 and 4.5 ± 3.8 mm Hg, respectively. The authors concluded, however, that due to extreme inter-individual variability, these manoeuvres did not improve PetCO_2_’s assessment of PaCO_2_. However, PetCO_2_ can identify the most severe hypercapnia and hypocapnia. A PetCO_2_ between 30 and 35 mm Hg most often corresponds to normocapnia (35 to 45 mm Hg).

Several pathophysiological mechanisms could be involved to explain the effectiveness of our manoeuvre, as follows.

First, maintaining high pressure in the airways for a period of time allows some collapsed alveoli to open, homogenizing the distribution of the ventilation/perfusion ratio. The alveoli recruitment mechanism was evidenced by the greater decrease in the carbon dioxide gradient in the COVID group, demonstrated by a mean difference Δ(a-A)CO_2_ − Δ(a-et)CO_2_ of −12.2 vs. −7.5 mm Hg in the anaesthesia group. Several studies have shown that prolongation of the inspiratory time decreases alveolar dead-space [9,25]. Diffusion of CO_2_ is time-dependent, and this prolongation increases the time available for alveolar gas exchange.

Second, during apnoea, increased inspiratory time allows homogenisation of PCO_2_ in the alveoli and between the alveoli and blood. During prolonged apnoea, alveolar PCO_2_ approaches pulmonary venous blood PCO_2_ due to the absence of alveolar gas movement. This effect has certainly contributed to the reduction of CO_2_ gradients.

Under ARDS conditions, tidal volume was set to 6 mL/kg with a high respiratory rate to maintain alveolar ventilation and reduced expiratory time. This increased the consequences of inequalities in regional respiratory time constants. Hence, the PetCO_2_ value depended on short- and long-time constant alveoli gas mix. PACO_2_ tends toward the central venous PCO_2_ due to reduced expired volume of poorly ventilated alveoli. Setting the respiratory rate to 10 cycles per minute prior to each PACO_2_ measurement improved the expiration of long-time constant alveoli. The recruitment of high CO_2_-concentrated alveoli cumulated with the prolongation of expiration, inducing an increased expiratory CO_2_ peak, with PACO_2_ over-estimating the PaCO_2_ [26,27].

Late emptying of well-perfused alveoli with higher CO_2_ tensions and better overall ventilation/perfusion matching helped to reduce the gradient between PaCO_2_ and PetCO_2_, which became negative in some cases. Negative (a-et) PCO_2_ gradients have been reported in infants and children, in pregnant patients, and during exercise [11,26]. This late mechanism may play an important role.

Fletcher and Jonson have studied the Vd/Vt ratio and P(a-et)CO_2_ gradient at two levels of Vt (450 and 750 mL) in anaesthetised patients [28]. Increasing Vt and decreasing the respiratory frequency did not change the airway dead-space but decreased the alveolar dead-space ratio. This improvement with increasing Vt was attributed to beneficial effects on gas distribution and diffusion time. At large Vt, the P(a-et)CO_2_ gradient decreased from 4.5 to 2.5 mm Hg, with a negative gradient in some patients. It is difficult to distinguish between what amounts to an increase in inspiratory time and expiratory time. Increased inspiratory time improves the distribution of the ventilation/perfusion ratio, and increased expiratory time decreases alveolar dead-space.

Third, PetCO_2_ and PACO_2_ are measured by computer analysis of the highest point reached by the capnogram curve at the end of the expiration. The overall performance (response time) of the capnograph including the sample line may be insufficient when high respiratory frequencies are used [24,29]. It is possible that some PetCO_2_ values may have been underestimated due to insufficient capnograph response time relative to the respiratory rate used. Therefore, the 4 s extended expiration time reduced the expiratory slope and thus reduced the bias in the computer analysis of PetCO_2_.

Our study also had some limitations. First, the lack of hypocapnic patients does not allow us to draw conclusions about the capacity of the measurement to detect a PaCO_2_ less than 35 mm Hg under such conditions. Second, some measurements were suspected to have been collected under not completely apnoeic conditions. However, the accuracy of the Δ(A-a)CO_2_ was relatively unaffected by the inclusion or not of these measures in the Bland–Altman diagram.

Initially, the data analysis protocol did not anticipate how to deal with the PACO_2_ values obtained using both the inspiratory pause and recruitment methods. We chose to only consider the first of the two measures, hypothesising that the residual effect of the first might bias the interpretation of the second.

According to the Berlin definition [10], there was no severe ARDS in our study, and patients were not haemodynamically unstable. We cannot draw conclusions about the effectiveness of our methods in patients with more severe lung diseases.

To conclude, measuring PCO_2_ after a prolonged inspiration and expiration improves the estimation of PaCO_2_ from PetCO_2_ and the identification of hypercapnic patients mechanically ventilated during anaesthesia and for COVID-related ARDS. Measuring end-tidal PCO_2_ after prolonged inspiratory time reduced the PaCO_2_–PetCO_2_ gradient to the point of obtaining values close to PaCO_2_. This non-invasive measure seems particularly interesting when using small Vt in the protected ventilation. However, further studies are needed to explore its usefulness in other conditions, such as in brain-injured patients, severe ARDS, and/or hypocapnic patients.

## Figures and Tables

**Table 1:** Patient characteristics.

	COVID	Anaesthesia
Measure (*n*)	35	32
% male (M/F ratio)	0.29 (2/5)	0.41 (11/16)
Mean Age (years) (range)	65 (59–73)	59 (23–78)
Ventilator (*n*)		
Evita	20 (5)	0
Perseus	12 (2)	30 (24)
Zeus	0	5 (3)
Type of manoeuvre (*n*)		
Increased inspiratory pause	35	27
Recruitment	0	5
Heart rate (bpm) (SD)	82 (13)	78 (13)
MAP (mm Hg) (SD)	81 (13)	78 (14)
Norepinephrine (mg h^−1^)	0.21 (0.46)	0.24 (0.88)
Temperature (°C) (SD)	37 (0.69)	36 (0.71)
PaCO_2_ (mm Hg) (SD)	49.3 (11)	42.2 (5.0) *
PaCO_2_ (*n*)		
≤35 mm Hg	1	3
35 to ≤45 mm Hg	13	19
≥45 mm Hg	21	10
Compliance (mL cm H_2_O^−1^)	24 (9.8)	72 (86) *
PaO_2_/FiO_2_ (mm Hg) (SD)	224 (53)	367 (110) *

MAP, mean arterial pressure; PaCO_2_, arterial carbon dioxide pressure; compliance = Vt/(Pplate-PEP): Vt, tidal volume; Pplate, plate pressure; PEP, positive end pressure. *, *p* < 0.05 versus COVID group.

**Table 2:** Mean PCO_2_ (mm Hg), and Student’s t-test for comparison of paired values in the COVID group, the anaesthesia group, and the combined groups.

Gradient	COVID			Anaesthesia			Combined		
	Mean	SD	*p*-Value	Mean	SD	*p*-Value	Mean	SD	*p*-Value
PaCO_2_	49	11		42	5.0		46	5.1	
PetCO_2_	39	9		37	4.0		38	7.3	
PACO_2_	51	11		44	4.7		46	4.6	
D(a-et)CO_2_	+11	5.7	<0.001	+5.7	3.4	<0.001	+8.2	5.4	<0.001
D(a-A)CO_2_	−1.2	3.3	0.043	−1.9	3.3	<0.003	−1.5	3.3	<0.001

## Data Availability

Data available on demand (arthur.salome1995@gmail.com).
